# Retracted: High Expression of PTGR1 Promotes NSCLC Cell Growth via Positive Regulation of Cyclin-Dependent Protein Kinase Complex

**DOI:** 10.1155/2017/7640820

**Published:** 2017-08-28

**Authors:** BioMed Research International

BioMed Research International has retracted the article titled “High Expression of PTGR1 Promotes NSCLC Cell Growth via Positive Regulation of Cyclin-Dependent Protein Kinase Complex” [1]. A series of very similar articles on shRNA and cancer cell lines was identified by Byrne and Labbé [2], and the intertextual distance between this article and an article in the series [3] is lower than expected by chance. The following concerns were found:

- The supposed nontargeting control shRNA sequence, 5 GCGGAGGGTTTGAAAGAATATCTCGAGATATTCTTTCAAACCCTCCGCTTTTTT-3, targets TPD52L2 (NM_199360). The same sequence was used as a nontargeting control in other articles identified by Byrne and Labbé.
- The article is very similar in methods and structure to two other studies that also use this incorrect sequence [4, 5].

The authors did not respond to requests for comment.
